# Increasing uptake of evidence-based screening services though a community health worker-delivered multimodality program: study protocol for a randomized pragmatic trial

**DOI:** 10.1186/s13063-020-4213-7

**Published:** 2020-04-29

**Authors:** Olveen Carrasquillo, Julia Seay, Vasanti Jhaveri, Timothy Long, Sonjia Kenya, Emmanuel Thomas, Daniel Sussman, Dinah Trevil, Tulay Koru-Sengul, Erin Kobetz

**Affiliations:** 1 Sylvester Comprehensive Cancer Center, Clinical Research Building, University of Miami, Miller School of Medicine, 1120 NW 14th Street, Miami, FL 33136 USA; 2 Department of Medicine, University of Miami Miller School of Medicine, 1120 NW 14th Street, Miami, FL 33136 USA; 3 Department of Public Health Sciences, University of Miami Miller School of Medicine, 1120 NW 14th Street, Miami, FL 33136 USA; 4 Health Choice Network, 9064 N.W. 13 Terrace, Miami, FL 33172 USA

**Keywords:** Cervical cancer, Colorectal cancer, Hepatitis C, HIV, HPV, Haitian, Hispanic, Immigrant, Screening

## Abstract

**Background:**

Underserved ethnic minority populations experience significant disparities in HIV, hepatitis C virus (HCV), colorectal cancer (CRC), and cervical cancer incidence and mortality. Much of the excess burden of these diseases among underserved communities is due to lack of preventive care, including screening. Barriers to disease screening include low awareness, lack of access to care and health insurance, and cultural beliefs regarding disease prevention. Our current trial aims to examine community health worker (CHW)-delivered, home-based multi-modality screening for HIV, HCV, CRC, and cervical cancer simultaneously.

**Design:**

We are conducting a randomized pragmatic trial among 900 Haitian, Hispanic, and African-American participants from diverse underserved communities in South Florida. People between the ages of 50 and 65 who have not had appropriate HIV, HCV, CRC, and cervical cancer screening per United States Preventive Services Task Force (USPSTF) recommendations are eligible for the study. Participants are recruited by CHWs and complete a structured interview to assess multilevel determinants of disease risk. Participants are then randomized to receive HIV, HCV, CRC, and cervical cancer screening via navigation to care by a CHW (Group 1) or via CHW-delivered home-based screening (Group 2). The primary outcome is completion of screening for each of these diseases within 6 months post-enrollment.

**Discussion:**

Our trial is among the first to examine the effectiveness of a CHW-delivered, multimodality, home-based disease-screening approach. If found to be effective, this approach may represent a cost-effective strategy for disease screening within underserved and underscreened minority groups.

**Trial registration:**

Clinical Trials.gov # NCT02970136, registered November 21, 2016.

## Background and rationale

Minority, low income, and underinsured communities are at excess risk of morbidity and mortality from HIV, hepatitis C virus (HCV), colorectal cancer (CRC), and cervical cancer [1–10]. Lack of screening and/or early detection contributes greatly to this excess risk. Barriers to appropriate screening include poverty, language difficulties, limited access to health care (including, but not limited to, lack of health insurance), low health literacy, lack of knowledge about various diseases and the importance of early detection, disease fatalism or the belief that disease implies death, and cultural norms about health and disease prevention. Alongside these barriers, historically and currently these communities have also been subject to discrimination and anti-immigrant policies, which further complicates the issue of accessing routine screening. 

Alternative screening strategies, such as screening via home-based and point of care tests, may address some of these barriers. Home-based tests for HIV, HCV, CRC, and cervical cancer are currently available, but not yet widely used.^i-iv^ With these, individuals can test themselves outside a formal clinical setting. Both within and outside the USA, randomized studies of home-based disease screening (most using mailed kits) have shown increased rates of screening [11, 12]. These screenings are most effective when delivered by a community health worker (CHW) who is knowledgeable regarding community values and norms [12].

We have recently completed two randomized trials of CHW-delivered, home-based cervical cancer screening among Haitian, Hispanic, and African American women living in South Florida [13, 14]. These studies revealed that CHW-delivered, home-based screening was superior in increasing cervical cancer screening uptake. We now seek to examine whether additional home-based screening modalities can be delivered together to improve not only cervical cancer screening but also screening for HIV, HCV, and CRC among racial/ethnic minorities.

### Aims and objectives

Our specific aim is to determine if a strategy in which CHWs deliver multimodality, home-based screenings results in an increase in the proportion of participants screened for all four conditions (HIV, HCV, CRC, and cervical cancer) as compared to a strategy in which patients are navigated to primary care by a CHW at one of our participating health centers. Our study will have over 95% power to examine our primary hypothesis that the CHW led multimodality screening strategy will result in a 15 percentage point increase in participants who are up to date in screening for these four conditions (three for men) as compared to a strategy of linkage to primary care. Secondary analysis will examine increases in screening for each of the four conditions individually. Subgroup analysis will include examining outcomes by race/ethnicity and by gender.

## Methods

### Trial design/setting

We are recruiting 900 participants from various underserved communities in Miami-Dade County. Of the 2.8 million county residents, 69% are Hispanic and 18% are Black [15]. Further, whereas 30 years ago, Cubans made up 80% of Miami Hispanics, over the last decades a rapid influx of immigrants from most of Latin America now make Miami as the most diverse Latino community in the USA. In 2018, 52%Latinos were Cuban, 20%^17^ were South American, 15% were Central American, and 9% were Puerto Rican or Dominican[15]. South Florida’s Black population is also diverse, and Miami has the largest population of Black immigrants. Nationally, the Black immigrant population has quadrupled and they will represent nearly 20% of Black Americans in 50 years. However, at present, more than a third of Black residents in Miami are foreign-born. This includes the 484,000 Haitians living in Florida, who represent 47% of all US Haitians [16]. For the current study, we are focusing recruitment primarily in three communities: Little Haiti (predominantly Haitian), Hialeah (predominantly Hispanic), and South-Dade (racially/ethnically mixed).

#### Conceptual approach

Our conceptual approach to addressing disparities in cancer control is informed by Social Ecological theory, which emphasizes the importance of, and interaction between, larger systemic factors (relationships, communities, culture, etc.) and individual factors in determining health behavior [17]. Thus our approach aims to address the aforementioned barriers to cancer screening not only on the individual level, but also on the larger systemic, cultural, and community levels. To successfully address cancer control from a socioecological perspective, our study utilized a community-based participatory research (CBPR) approach. CBPR addresses cancer control at multiple levels of influence via the engagement of key community stakeholders [9]. These stakeholders advise and inform the development of interventions tailored specifically to meet unique community need, which facilitates the implementation at both the individual and community levels. Our community partners, including federally qualified health centers (FQHCs), community-based organizations, community leaders, as well as members of the target communities, provide critical input regarding all aspects of study design and implementation within the current study.

### CHW training

As with prior studies, our community outreach, recruitment, and intervention approaches are led by CHWs. Our study team includes three CHWs all from our target communities who are bilingual in either English and Spanish or English and Haitian Creole. All three are certified CHWs by the Florida Certification Bureau and each has over 5 years of experience as a CHW. All three have completed the University of Miami’s required research training in human subject research as well as formal CHW research training using modules developed by our study team. They also received additional study-specific training from our academic research team, organized into three modules: (1) health education on each of the four conditions, (2) specimen collection procedures, and (3) study data collection procedures. Each of these sessions was interactive and hands-on, and CHWs were able to practice and receive feedback on their data collection and study procedure skills.

### Eligibility

#### Inclusion criteria

To be eligible individuals must be: (a) Haitian, Hispanic, or Black, (b) report not having had appropriate screening for at least one of the following HIV, HCV, CRC, and cervical cancer (as per 2017 United States Preventive Services Task Force (USPSTF) guidelines: never had a HIV test; never had a HCV test; not had a colonoscopy in last 10 years and/or stool-based test in the last year; and not had a Pap smear within the past 3 years), and (c) age 50–65 years. Each preventive test has different age and other eligibility criteria. For example, cervical cancer screening is recommended for women ages 21–65, CRC is recommended from ages 50–75, and HCV is recommended for persons born between the years of 1945 and 1965. After consultation with our community partners, we selected this simplified age criterion (50–65 years), which maximizes the number of tests for which participants would be potentially eligible (three for men, four for women).

#### Exclusion criteria

Individuals are not included in the study if they: (1) plan to move out of the county during the next 6 months, (2) have current or prior enrollment (5 years) in any research study that involved screening for these conditions, (3) are unable to consent, and/or (4) are currently pregnant.

### Recruitment

Trial recruitment occurs via convenience sampling at community-based locations such as markets, churches, botanicas, and health clinics, as well as via the social networks of our CHWs and community partners. The CHWs approach potentially eligible individuals, screen for eligibility, and explain the study. Eligible and interested individuals are asked to provide their contact information, and CHWs will try up to ten times to contact eligible individuals to schedule them for informed consent and study intake. Participants who agree to participate complete a 30‑minute intake interview in their home or location of their preference. The CHWs work closely with our two bilingual research associates (RAs) to set up these visits during which informed consent and study intake occur. Our RAs are master’s level highly trained research professionals. Upon completion of the intake interview, participants are randomized into one of two interventions.

### Allocation of trial interventions

Following the study intake, participants are randomized to one of the two study interventions by the study biostatistician, using REDCap’s randomization module. The module randomizes study participants electronically, and randomizes within study sites to account for nesting. The CHWs are notified via email of the assignment of participants following the randomization, and will follow up with participants within a week to inform them of their assignment, and to schedule a home visit (if the participant is in the home-based testing arm). Notably, while participants and CHWs are aware of group assignment, the community health educator (CHE), who collects study outcome data, is blinded to participant assignment.

#### Intervention Group 1—CHW-delivered navigation to primary care (PC) for disease screening

For participants assigned to Group 1, CHWs work closely with their FQHC supervisor to develop tailored navigation approaches appropriate for each person (consistent with the pragmatic trial design). First, they contact participants to assess which preventive services they are eligible for using a checklist based on the above criteria. For participants who already have a primary care provider (PCP), CHWs encourage such persons to follow up with their provider for needed tests. If they do not have a PCP or prefer not to go to their PCP for such testing, the preferred approach is to navigate participants to primary care services at local FQHCs where providers as part of routine care evaluate patients for needed preventive services. CHWs emphasize that while they are following a broad set of screening guidelines, only a PCP will know which preventive services are needed for each person based on their unique circumstance and risk profiles. As health center outreach workers, CHWs do not limit the scope of work to the four screening services. They also help link study subjects with other services offered by the centers (e.g. facilitated enrollment in health insurance plans, immigrant and refugee programs, behavioral health, and programs for people with diabetes), as needed. Participants may also prefer to obtain these services from other locations such as Project Screen (free cervical cancer screening for women age > 50), locations providing anonymous HIV and HCV testing, or health fairs sponsored by health centers or the University of Miami. In the third year of the study, the University of Miami began a separate, community-based cancer screening program using a mobile van that offers these screening services for free. On average, the vehicle goes to each of these communities at least once a month. Thus, we included the mobile van as an additional screening referral option for study participants in this group.

#### Intervention Group 2—CHW-delivered, home-based testing (HBT)

The CHWs develop tailored approaches appropriate for each participant in Group 2. First, CHWs use a checklist (as described above) to help determine which screening tests are needed. Participants in Group 2 are offered home-based testing. For these participants, there are various strategies CHWs may use. First, the CHW and the participant select a place of mutual agreement to conduct the needed screenings. Most typically, it will be at the participant’s home, although it can be at another community location. At the intervention visit the CHWs perform HCV and HIV screenings and provide post-test counselling. For the cervical cancer screening, they instruct participants how to do human papilloma virus (HPV) self-sampling. Participants are offered HPV self-sampling at the intervention visit, and typically provide the self-collected specimen to the CHW during the intervention visit. If for some reason the participant cannot complete the HPV self-sampling during the intervention visit, the CHWs will leave the kit with the participant and the participant can send the sample via mail. Persons needing a fecal immunochemical test (FIT) receive instruction on how to do the test and are given the kits, which they mail back. For mailed kits, the CHWs conduct follow-up phone calls encouraging participants to mail the kits back (FIT, HPV). CHWs also call participants with the results of the home-based testing, and, if needed, do home visits for post-test counselling. All participants who elect to do home-based testing also receive a letter explaining their results which they can provide PCPs. All participants who elect to do home-based testing are still urged to follow-up with a PCP for routine primary care. CHWs explain to participants that the PCP can not only review the letter describing the tests the CHW provided to the participants but also assess for other additional screening tests or follow-up treatments the participants may need.

### Home-based screening tests

#### HIV screening: OraQuick HIV

Participants are screened for HIV 1(1/2) using oral swab testing: OraQuick® HIV (OraSure Technologies, Bethlehem, PA, USA). OraQuick Test detects HIV antibodies in the oral fluid. The test uses an oral swab to collect mucosal transudate by swabbing the gums. Participants can perform the test themselves or have the CHW (using appropriate blood-borne pathogen precautions) collect the swab for them. The CHWs received training and certification to meet the Florida Department of Health’s Minimum Standards for HIV Counselors. They were trained in OraQuick testing procedures as coordinated by a study co-investigator (SK) with extensive expertise in community-based HIV testing. Clients who test positive receive post-test counselling and are referred for free confirmatory testing at either one of the participating health centers of the South Florida AIDS network.

#### Hepatitis C screening: OraQuick HCV rapid antibody test

Participants are screened for HCV infection using a finger stick blood test, the OraQuick® HCV Rapid Antibody Test HCV (OraSure Technologies Inc., Bethlehem, PA, USA). This test has been shown to have a specificity of > 99.6%, which is nearly as specific as testing using venous blood. CHWs were trained on the procedures for HCV testing and post-test counseling by a co-investigator with extensive expertise in community-based HCV testing (ET). While patients can elect to do their own finger stick, in our prior HCV outreach programs, most participants have preferred for CHWs to do the finger stick. If a participant screens positive for HCV they will have the option of going to their PCP, a local FQHC, or the University of Miami’s Schiff Center for Liver Disease for confirmatory blood testing. For participants with positive confirmatory testing, the CHWs are available to explain and discuss in greater detail the serology results and the potential for complications for individuals living with HIV. The importance of screening for participants’ family members will also be emphasized. Participants are also advised that if their providers would like to place them on treatment, the CHWs can help find programs to cover the cost of the medication. For example, through a unique academic-industry partnership brokered by the University of Miami (UM)’s Schiff Center, the Center is able to provide free treatment for low-income, medically indigent patients who need HCV medications.

#### CRC screening: fecal immunochemical testing (FIT)

Participants are screened for CRC using home-based FIT testing. All CHWs are already experienced conducting community-based FIT screening from prior research studies and outreach initiatives [18]. CHWs have also been previously trained in FIT procedures including post-test counseling and the system to navigate patients for follow-up colonoscopy if needed. We use a Clinical Laboratory Improvement Amendments (CLIA)-approved FIT (OC-Auto Micro 80, Polymedco/Eiken, Cortlandt Manor, NY, USA). This FIT is a quantitative test with a cutoff for a positive, abnormal result of 100 ng hemoglobin/mL stool. Participants first receive education on the importance of CRC screening and how to properly perform the FIT test using educational materials from the American Cancer Society and the manufacturer. The study team translated these materials into Spanish and Haitian Creole using standard back-translation methods, and then tailored these materials to health literacy level of our study populations. Participants are provided with a FIT collection kit consisting of the collection device, instructions, and a mailer so that the sample may be sent to a central laboratory. CHWs subsequently make follow-up calls to reinforce education on proper collection and to ensure return of FIT specimens. Participant samples are mailed to a central UM laboratory using the mailers and self-addressed, stamped envelopes provided in the FIT kit. Individuals are asked to record the date of stool collection on the mailers, and the date of sample receipt by the laboratory will also be recorded. CHWs will deliver test results to participants by phone or in-person. If FIT results are normal, these patients will be instructed by the CHW they need to repeat CRC screening in 1 year. If the FIT test is positive, participants are advised that they need to undergo follow-up testing with colonoscopy. Participants can choose to follow-up with their own PCPs for colonoscopy, be referred to a local FQHC to arrange for such follow-up care, or have the CHWs assist with arranging follow-up. For example, the procedure can be done at the county’s local public hospital, Jackson Memorial Hospital. The CHWs are very familiar with navigating participants through the Jackson’s financial classification program for the medically indigent, so that they can get low-cost or free colonoscopies. The goal is to have the colonoscopy done within 90 days of the positive result. CHWs also provide education and participant reminders, ensuring proper preparation for colonoscopy and, if needed, helping arrange transportation to and from the colonoscopy appointment if necessary.

#### Cervical cancer screening: HPV self-sampling device

For home-based cervical cancer screening, CHWs will also continue using the same approach we have used in our prior studies [13, 14]. We use the Preventive Oncology International (POI)/National Institutes of Health (NIH) HPV self-sampling device. This device has been found to be of similar sensitivity to physician-collected specimens for high-risk HPV detection [19, 20]. The self-sampler includes a Dacron swab and a vial of ThinPrep. This self­sampler is similar in size to a regular tampon and very easy to use. The participant inserts the swab into her vagina until she meets resistance, which indicates the swab has reached her cervix. She then turns the swab three times to collect the sample, withdraws the swab, and then inserts the swab into the ThinPrep and stirs the swab 10 times. The CHWs refer those who screen positive to follow-up Pap smear and/or colposcopy at their local FQHC.

### Trial procedures 

#### Enrollment visit

During the enrollment visit, the CHE explains the study to the participants, answers participant questions, confirms participant eligibility, and obtains informed consent. Following informed consent, the CHE conducts an intake interview. Based on our experience with respondent fatigue as well as based on the recommendations of our community partners, we try to keep the intake interview to no more than 30 minutes in length. The interview includes questions on sociodemographic characteristics, previous experiences with disease screening for each of our four conditions, condition-specific disease knowledge, health beliefs and access to care. The intake interview (as well as informed consent and all other study measures), have been translated into Spanish and Haitian Creole by certified translators using validated back-translation methods.

#### Follow-up interview at 6 months

At 6 months, the CHW schedules each participant for a follow-up interview with a CHE who is blinded to study allocation. CHWs remind participants not to reveal their allocation to the CHE. Throughout the study, the CHEs (rather than the CHWs) conduct assessments to avoid bias. The follow-up interview includes some of the measures covered in the intake interview (screenings, health knowledge, access to care), as well as measures examining the acceptability of the intervention (for both study arms).

#### Participant timeline

**Fig. 1:**
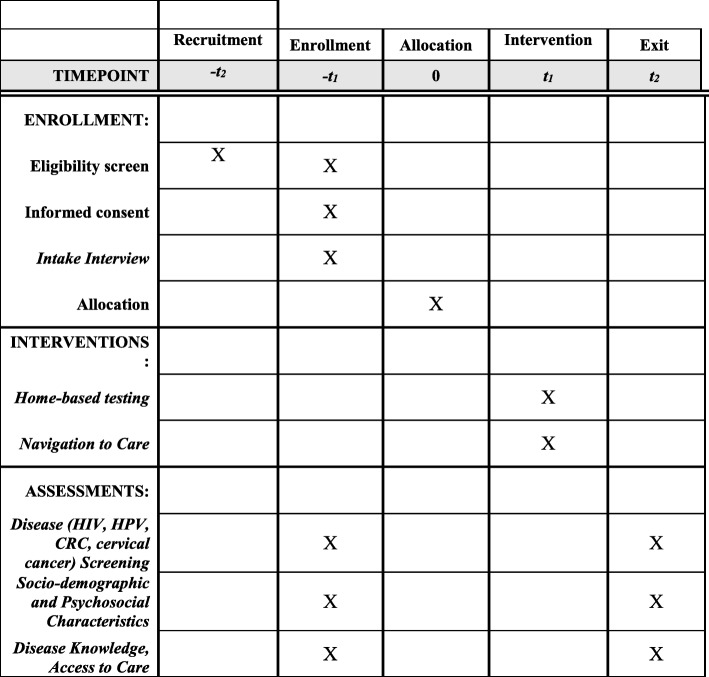
Schedule of enrollment, interventions, and assessments

The study is planned to be 60 months. Enrollment began in month 12 and will end by month 44. The recruitment goal is approximately 10 participants per month per community/study site. These targets and timelines are informed by our two previous trials within these communities (See Fig. 1).

### Outcome measures

#### Primary outcome

Our primary study outcome measure will be proportion of participants fully up to date on all their needed screenings for these four health conditions (three for men) at 6 months post-study enrollment. We will conduct intention-to-treat analysis based on study allocation. This outcome will be a binary (yes/no) variable. Participants in either group lost to follow-up at 6 months will be assumed not to have been screened in intention-to-treat analyses.

#### Secondary outcomes

As a secondary study outcome, we will examine each screening individually as a binary variable and look at the change in screening completion from baseline to 6-month follow-up in each arm of the study. For example, change in proportion of people up to date in screening for HCV in each arm. Another secondary outcome we will consider is the increase in median number of screenings done. For this endpoint, the possible ranges will be 0 to 4 for women (HIV, HCV, HPV, FIT) and 0 to 3 for men (HIV, HCV, FIT).

### Sample size calculation

The baseline screening rates in our sample size calculation rates are obtained from our feasibility studies and local data. The overall sample size was chosen to ensure sufficient power to detect clinically meaningful effects associated with a 15% point increase in proportion of additional screening done in the home-based arm versus primary care arm using two-sided test for differences in proportion. Although we plan to conduct intention-to-treat analyses, in which all women lost to follow-up will be considered “unscreened,” we will also conduct analyses with lost to follow-up participants excluded. For these analyses, we need to account for attrition (21% in our previous study). In addition, in our subgroup analysis where we look at each individual test, we set alpha at 1.25% instead of 5% to account for multiple testing. Even with a 25% attrition rate, a proposed total sample size of 900 participants (450 per arm and 150 per site) will give us over 95% power for our primary outcome and 90% power for the subgroup analysis.

### Fidelity

Intervention fidelity is assessed via weekly review of CHW encounter logs, which are documented every time the CHW has contact or attempts contact with a participant. Any issues or inconsistencies elucidated by this regular review are addressed and resolved at team meetings. In addition, we will assess participant knowledge of the four target conditions and screening following the intervention, which can help assess if disease education was administered effectively by the CHWs.

### Withdrawal of participants from study

Participants may request to be withdrawn from the study at any time by contacting the CHE. If a participant elects to withdraw, their reason for withdrawal will be recorded and their study data will be deleted.

### Data management

Study data will be collected and stored within the REDCap system, which is a web-based, secure, and HIPAA-compliant research management platform. REDCap fully supports several research processes for patient recruitment and scheduling, participant randomization, data entry and management, as well as data safety monitoring and adverse event reporting. A particular advantage of REDCap is that CHEs are immediately notified if fields are missing and/or if out or range values are entered. However, in case of loss of internet access, backup paper copies are provided, which would then be subsequently entered into REDCap. The data manager regularly screens the REDCap data for quality control, and any issues or inconsistencies are discussed with the CHEs. De-identified data is extracted from REDCap for data analysis.

### Statistical analysis

Prior to any analysis, data screening for accuracy will be conducted and will focus on several aspects. We will determine the amount, pattern, and randomness of missing data. Outliers will be identified observations that appear to be very high or very low, and decide how to proceed based on the presumed cause. We will also identify inconsistencies within a single variable and between variables. As there are various types of measurements, we will use various descriptive statistics to visualize the study data with the appropriate graphical displays. Initially, we will perform an explanatory data analysis; visually via graphics/plots such as scatter and box-whisker plots and numerically by descriptive statistics such as range, median, means, standard deviations for measurements taken on a continuous scale, and percentages, various types of cross-tabulations for measurements taken on a categorical scale. Corresponding confidence intervals for means and proportions will also be calculated. To have a better understanding of the relationships among different study measurements, parametric or non-parametric correlation coefficients, bivariate, and multidimensional cross-tabulations, scatter and box-whisker plots will be constructed. Prior to analyses, baseline differences of key covariates from the PC and HBT arm will be examined with respect to our primary and secondary outcomes, particularly those related to race, ethnicity, SES and education. We will also examine differences on key variables between subjects who completed the study and those lost to follow-up. Our primary analysis will consist of chi-squared testing for the differences in proportions among the PC versus HBT study arms. Several univariate and multivariate logistic regression models will be used to calculate odds ratios and corresponding 95% confidence intervals for exploring the result of the screening and the relation to various types of key study variables in addition to study arms. Two-way interaction terms between for various study variables and the study arms may need to be included in the models. Transformations of the continuous data in order to meet statistical assumptions may be undertaken if indicated. Type-1 error will be set to 5% (α = 0.05) for calculating confidence intervals and testing various hypotheses for the primary outcome. Adjustment of type-I error for multiple comparisons will be performed by controlling the family-wise type-I error rate at the 0.05 level. Standard diagnostic tools will be used to assess any model fit. All statistical analyses will be carried out using SAS® v9.4 for Windows (SAS Institute, Cary, NC, USA) and/or R-project for Windows.

#### Subgroup analyses

We plan to examine potential moderators of intervention effectiveness, including community/study site, ethnicity, education level, health insurance status, and acculturation. The interactions between these variables and study group assignment will be modeled and significant interactions will be decomposed and graphed.

#### Data monitoring

A Data Safety and Monitoring Board (DSMB) is required for all clinical trials at the University of Miami’s Sylvester Comprehensive Cancer Center (SCCC). These reviews are conducted by a standing committee (DSMC). The DSMC functions as an independent body, providing analysis of efficacy data and interim reviews of safety monitoring. DSMC membership consists of experienced investigators representing multiple specialties/disciplines. Sixty percent of current DSMC faculty members hold the rank of associate or full professor level and provide DSMC mentorship to assistant-level faculty members. The DSMC has full authority to require amendments to a study, suspend any study falling below DSMC standards, and/or provide recommendations to the Institutional Review Board (IRB) that the study be closed for lack of data integrity and/or patient safety reasons. Monitoring is conducted via monthly reports. Meetings are held on a monthly basis and trials with DSMC oversight are assessed based on the frequency associated with the assigned risk level. The DSMC report form provides an institutional mechanism for a protocol summary review and is the basis for reviewer evaluation of subject safety and trial conduct.

#### Adverse events

The study team will collect information and monitor any adverse events, including any injury incurred as a result of self-testing. If an adverse event occurs, participants will be immediately referred to appropriate medical care, and the study team will report the event to the principal investigators (PIs) and the IRB. Information regarding adverse events will be recorded in REDCap.

#### Protocol approvals and amendments

The current study was approved by the University of Miami IRB. There have been 13 minor amendments to update study documents, such as adding staff to the protocol (version date: October 15, 2019). Since the study began, there have been no changes in primary or secondary outcomes.

#### Informed consent

CHEs obtain informed consent in the participant’s preferred language (the informed consent document available in English, Spanish, and Haitian Creole, and CHEs are bilingual), and will answer all participant questions and ensure participant comprehension prior to obtaining signatures. For participants with limited literacy, the CHEs will read the consent verbatim with a witness present to ensure no bias enters the consent process.

#### Confidentiality

To protect confidentiality, all participants are assigned a study ID number and only this number (no identifying information) is included on study assessments. Datasets will be de-identified prior to analysis, and all study data will be stored on secure servers. In addition, any paper files are stored in locked file cabinets in secure office spaces.

#### Financial interests

All of the study team members, as well as the study staff, have no financial interests to disclose.

#### Access to data

Regarding access to data, only the data manager and study biostatistician will have access to raw data files. If necessary, the data manager may share cleaned and de-identified data with other study staff for ongoing data analysis and monitoring such as for the creation of tables and charts. Investigators outside the study may request access to de-identified datasets through the study PIs.

#### Post-trial care

As noted above, all participants who screen positive for any of the four conditions are navigated by the CHW to free or low-cost follow-up care, and CHWs follow-up with these participants to ensure appointments are made, and to address any barriers to obtaining follow-up care. Additionally, regardless of study allocation, any participants who have completed the study and remain unscreened at the 6-month follow-up assessment are offered either home-based screening or navigation to a provider for screening by the CHW, based on participant preference. However, to avoid any bias or interference with intervention completion, participants are not previously informed that the screenings will be offered at the conclusion of the study. This post-study testing will not be part of data analysis.

#### Dissemination

While we will share our findings through academic channels such as journals and conferences, as consistent with CBPR, we will also share our results with the study communities. To aid with this process, community partners will help disseminate our study findings through culturally appropriate communication channels, such as organizing a series of community forums.

## Discussion

Our project addresses the excess burden of HIV, HCV, CRC, and cervical cancer experienced by Little Haiti, Hialeah, and South Dade, three medically underserved communities within the Miami metropolitan area. Using a CBPR approach, we will determine the effectiveness of navigation to primary care for screening versus CHW-delivered, home-based, multimodality screening among 900 individuals from our target communities. Study findings will allow us to optimize this for disease screening in real-world settings.

There are several study limitations that should be considered. There is only one CHW per community, which impedes our ability to determine whether potential differences in intervention effectiveness by community are due to community or CHW-related factors. We apply regular monitoring and quality control to maximize fidelity among study CHWs. Moreover, the CHW encounter logs may be used to better understand the influence of CHW-related factors on study outcomes. Our results also may not be generalizable outside of our target communities, as the current intervention approaches are heavily tailored based on community partner input. While this approach is a valid method of addressing health disparity within these specific communities, the effectiveness of multi-modality home-based testing would likely need to be re-examined if implemented outside of our target communities. Lastly, our primary outcome is based on self-report. However, self-reported data may overestimate the proportion of persons who obtained the screenings. For participants in the HBT group who choose home testing, we will have objective lab data to validate their self-reported data. However, if participants in the PC group overestimate their screenings, it could bias our results towards the null hypothesis.

## Trial status

The first trial participant was enrolled on June 26, 2017. As of January 2020 we have enrolled 705 of our target 900 participants in the study. The trial is projected to end in December 31, 2020. The current protocol is version #5, November 19 2018.

## Data Availability

Not applicable.

## Abbreviations

CBPR: Community-based participatory research
CHE: Community health educator
CHW: Community health worker
CRC: Colorectal cancer
DSMB: Data Safety and Monitoring Board
FIT: Fecal immunochemical test
FQHC: Federally qualified health center
HBT: Home-based testing
HCV: Hepatitis C virus
HPV: Human papilloma virus
IRB: Institutional Review Board
PC: Primary care
PCP: Primary care provider
PI: Principal investigator
RA: Research assistant
UM: University of Miami
USPSTF: United States Preventive Services Task Force
